# Mitochondrial Cyclosporine A-Independent Palmitate/Ca^2+^-Induced Permeability Transition Pore (PA-mPT Pore) and Its Role in Mitochondrial Function and Protection against Calcium Overload and Glutamate Toxicity

**DOI:** 10.3390/cells10010125

**Published:** 2021-01-11

**Authors:** Galina D. Mironova, Evgeny V. Pavlov

**Affiliations:** 1 Institute of Theoretical and Experimental Biophysics, RAS, Pushchino, 142290 Moscow, Russia; 2 Department of Molecular Pathobiology, College of Dentistry, New York University, New York, NY 10010, USA; ep37@nyu.edu

**Keywords:** mitochondria, palmitic acid, palmitate/Ca^2+^-induced permeability transition pore, calcium overload, glutamate toxicity

## Abstract

A sharp increase in the permeability of the mitochondrial inner membrane known as mitochondrial permeability transition (or mPT) occurs in mitochondria under the conditions of Ca^2+^ and ROS stress. Permeability transition can proceed through several mechanisms. The most common mechanism of mPT is based on the opening of a cyclosporine A (CSA)-sensitive protein channel in the inner membrane. In addition to the CSA-sensitive pathway, mPT can occur through the transient opening of lipid pores, emerging in the process of formation of palmitate/Ca^2+^ complexes. This pathway is independent of CSA and likely plays a protective role against Ca^2+^ and ROS toxicity. The review considers molecular mechanisms of formation and regulation of the palmitate/Ca^2+^-induced pores, which we designate as PA-mPT to distinguish it from the classical CSA-sensitive mPT. In the paper, we discuss conditions of its opening in the biological membranes, as well as its role in the physiological and pathophysiological processes. Additionally, we summarize data that indicate the involvement of PA-mPT in the protection of mitochondria against calcium overload and glutamate-induced degradation in neurons.

## 1. Introduction

Ca^2+^ is an ion involved in the regulation of many processes within living organisms and cells, including muscle contraction, neurotransmission, regulation of energy metabolism, and many others. The key regulatory role of Ca^2+^ is linked to its ability to be selectively redistributed across biological membranes via tightly regulated selective channels, exchangers, and pumps in response to specific stimuli. In addition to its regulatory role in physiological processes, Ca^2+^ plays a critical role as a mediator of stress-induced cell death. In many pathologies, disorders of ion homeostasis lead to a rise of the Ca^2+^ concentration in the cytoplasm and mitochondria, which eventually triggers a sharp increase in the permeability of mitochondrial membranes and cell death.

Under stress conditions, the Ca^2+^-induced increase in the permeability of the inner mitochondrial membrane is caused by the opening of a large protein channel, the so called mitochondrial permeability transition pore (or mPTP). Many laboratories worldwide extensively study mPTP (see [1,2,3] for recent reviews). Traditionally, mPTP is described in the literature as a large pore which is located in the inner mitochondrial membrane and allows passage of molecules up to 1.5 kDa in size. The key inducers of mPTP are Ca^2+^ and ROS, but it can also be affected and modulated by many other agents. A specific feature of mPTP is its sensitivity to cyclosporine A (CSA). In most cases, mPTP opening causes depolarization of mitochondria and cell death. On the other hand, mPTP has also been suggested to play an important physiological role—in cases when it is activated in a specific mode: either low-conductance or transient (flickering) [4,5]. The molecular architecture of mPTP is not completely understood, but it is generally agreed that mPTP has a protein nature and its opening can be mediated by ATP synthase and adenine nucleotide translocase [6,7,8,9]. The specific contribution of these proteins in the opening of mPTP is still debated though [10,11].

The research done by Prof. Mironova’s group over the past two decades suggests that the opening of CSA-sensitive mPTP is not the only mechanism by which mitochondrial membrane can be permeabilized under the conditions of Ca^2+^ stress. There may exist a fundamentally different mechanism which, in contrast to the classical one, is not CSA-sensitive and is associated with a “lipidic”, rather than “protein”, pore. The mechanism is based on the formation of complexes between anions of long-chain saturated fatty acids (mainly, palmitic acid, PA) and Ca^2+^, which takes place on the matrix side of the inner mitochondrial membrane and is accompanied by the appearance of transient lipid pores (PA-mPT pores). The central goal of this review is to discuss properties and physiological relevance of PA-mPT. Here, we shall survey the existing evidence that PA-mPT can function to prevent Ca^2+^ overload of mitochondria, playing a key role in the protection of the organelles against glutamate toxicity—and thus can be considered the “pore of life”.

## 2. Saturated Fatty Acids as Inducers of Membrane Permeabilization

The studies on the involvement of fatty acids in the mitochondrial permeability transition began in the mid-90’s of the past century. Initially, we discovered that the lipid fraction of the ethanolic mitochondrial extract induced an ion channel activity in planar lipid bilayers (black lipid membranes, BLM) [12]. Further purification and analysis of the fraction revealed that it contained some kind of a “Ca^2+^ sensor”, a protein-free component with high affinity for Ca^2+^ (K_d_ in the sub-µM range). The affinity further increased at alkaline pH (pH = 8.0), and reconstitution of the component into BLM enabled the formation of non-selective ion channels upon the addition of Ca^2+^ [13,14]. We also found that the component was predominantly located in the mitochondrial contact sites, the membrane’s regions enriched with transport proteins [13,14]. 

Further examination of the purified hydrophobic Ca^2+^-binding component revealed that it was mostly composed of long-chain saturated fatty acids, predominantly palmitic and stearic. Later, we demonstrated that synthetic palmitic and stearic acids had roughly the same Ca^2+^ affinity as the hydrophobic mitochondrial extract. At the same time, other hydrophobic compounds available in native membranes (including unsaturated fatty acids, phospholipids, and cardiolipin) had Ca^2+^ affinity being 1–2 orders of magnitude lower as compared to the affinity of palmitic and stearic acids (Table 1). Thus, we concluded that the Ca^2+^-binding properties of the hydrophobic mitochondrial extract were determined by the presence of stearic and palmitic acids [13]. 

**Figure 1:**
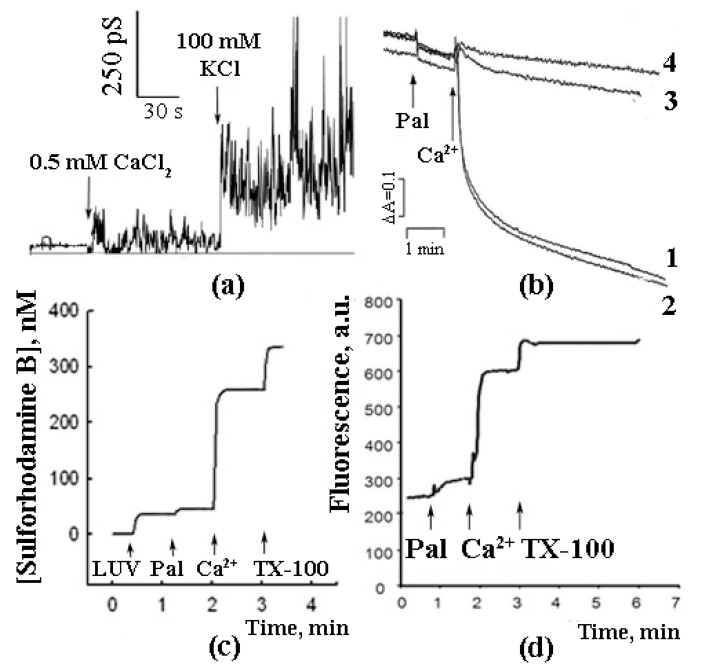
Palmitic acid can induce Ca^2+^-dependent permeabilization of artificial and biological membranes. (**a**) Planar lipid bilayer, PA 0.5%, membrane potential 100 mV. (**b**) Mitochondrial swelling: 1, 3, 4—no CSA, 2–1 µM CSA. Additions: (1, 2) 15 µM PA and 30 µM Ca^2+^, (3) 15 µM oleic acid (OA) and 30 µM Ca^2+^, (4) 15 µM brom-palmitic acid and 30 µM Ca^2+^. (**c**) Artificial liposcheme 50 µM PA and 1 mM Ca^2+^. (**d**) Plasmalemmal membranes of erythrocytes, release of the fluorescent probe sulforhodamine B from the erythrocytes induced by the addition of 50 µM PA and 1 mM Ca^2+^. Typical curves are shown (*n* = 6).

As shown in further experiments, the reconstitution of synthetic palmitic and stearic acids into BLM and the subsequent addition of Ca^2+^ induced a non-specific ionic conductance, which, in some cases, was accompanied by oscillations of ion currents (Figure 1a) [15]. A similar effect (Ca^2+^-induced permeabilization of membranes containing palmitic and stearic acids) was observed in the experiments with liposomes composed of phospholipids (Figure 1c) [15,16]. Palmitic and stearic acids were also found to cause a Ca^2+^-dependent permeability transition in the membranes of mitochondria (Figure 1b) isolated from various tissues (liver, heart, and brain) and in the membranes of erythrocytes (Figure 1d). It should be emphasized that, in case of mitochondrial membranes, the effect was CSA-independent, suggesting that the mechanism of membrane permeabilization was different from that of classical mPTP, which is known to be inhibited by CSA [17,18].

**Figure 2:**
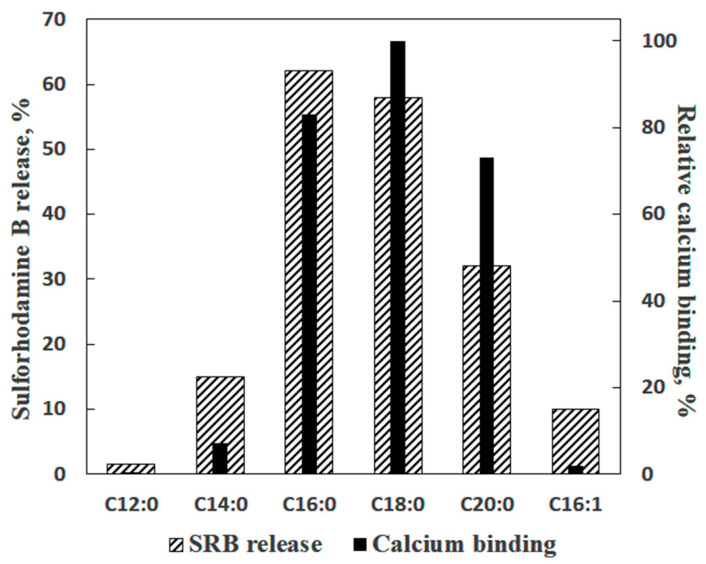
Ca^2+^-induced permeabilization of lecithin liposomes containing various fatty acids in comparison with the ability of fatty acids to bind Ca^2+^ ions. The data on the Ca^2+^-binding ability of fatty acids were taken from Table 1. Additions: 30 µM of a fatty acid and 1 mM Ca^2+^. The media contained 10 mM Tris-HCl (pH 8.5), 50 µM EGTA (ethylene glycol-bis(β-aminoethyl ether)-*N*,*N*,*N*′,*N*′-tetraacetic acid), and 40 µM KCl (*n* = 4).

In the experiments with liposomes, membrane permeabilization was monitored by measuring the release of sulforhodamine B (SRB), a membrane-impermeable fluorescent dye. The amount of SRB released from liposomes was proportional to the amounts of palmitic acid (PA) and Ca^2+^ added [16]. The release of SRB occurred instantaneously and stopped abruptly (Figure 1b). The pH optimum of the effect was in the same range (pH = 8.0) as in the case of formation of PA/Ca^2+^ complexes [13]. Testing various fatty acids in these experiments revealed a pattern similar to that observed previously in the Ca^2+^ binding assays: (1) a bell-shaped dependence of the effect on the length of the hydrocarbon chain in the series of saturated fatty acids; (2) the maximal effect for palmitic and stearic acids, and (3) much lower efficiency of unsaturated fatty acids, such as linoleic acid (Figure 2).

The experiments with liposomes also showed that PA-containing vesicles could be permeabilized by other divalent cations (Ba^2+^, Mn^2+^, Ni^2+^, Co^2+^, and Sr^2+^, in the descending order). An “outlier” was Mg^2+^, whose effect was much lower than that of other divalent cations (twice as low as the effect of Sr^2+^ and more than thrice as low as the effect of Ca^2+^). Additionally, we confirmed, using fluorescent correlative spectroscopy, that SRB release was not caused by vesicle micellization and, therefore, should be attributed to the formation of pores in the liposomal membranes [16].

## 3. Is PA-mPT Based on a General Mechanism of PA/Ca^2+^-Induced Permeabilization of Lipid Membranes?

As can be seen in Figure 1, the combined effect of PA and Ca^2+^ (i.e., their ability to permeabilize the lipid bilayer) was demonstrated on both artificial and biological (mitochondrial and erythrocytic) membranes. 

**Figure 3:**
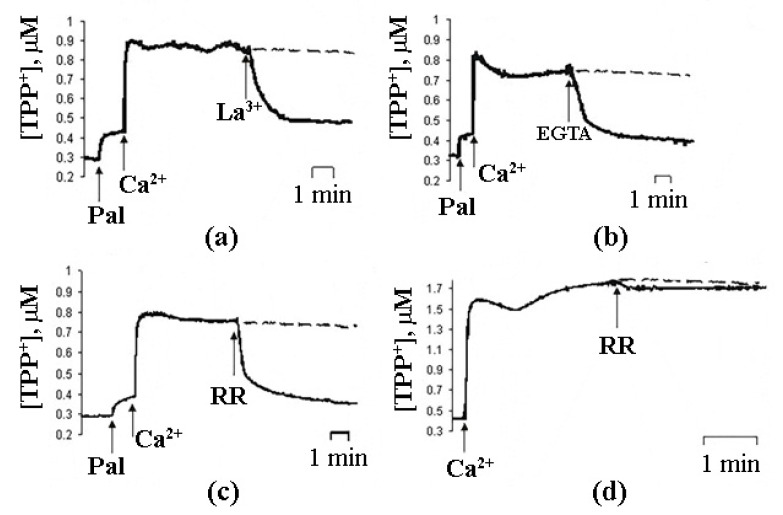
The effects of 5 µM La^3+^ (**a**), 1 mM EGTA (**b**), and 1 µM ruthenium red (RR) (**c**,**d**) on the changes in ψ induced by the opening of PA-mPT (**a**–**c**) and mPT (**d**). The incubation medium constable 210 mM mannitol, 70 mM sucrose, 5 mM succinate, 5 µM EGTA, 1 µM rotenone, and 10 mM Hepes/KOH (pH 7.4). Additions in (**a**–**c**): Pal (30 nmol/mg protein), Ca^2+^ (70 nmol/mg protein) and 1 µM CsA; in (**d**): Ca^2+^ (200 nmol/mg protein) and 1 mM Pi. Dashed line, control (without RR). Typical traces are shown (*n* = 7). Adapted from Mironova et al. J. Bioenerg. Biomembr. 39 (2007): 167–174, doi:10.1007/s10863-007-9079-9.

In case of intact mitochondria, the addition of PA alone did not produce any detectable effect, while the subsequent addition of Ca^2+^ induced a high-amplitude swelling of the organelles, indicating a transition in the permeability of the inner mitochondrial membrane to osmotic agents. This effect was not inhibited by CsA (Figure 1b) [13,17,19]. In the presence of PA and Ca^2+^, mitochondria became slightly depolarized but were able to maintain their function for quite a long time. We hypothesized that this phenomenological picture is characteristic of a flickering mode of permeability transition. The addition of EGTA or ruthenium red (i.e., the removal of Ca^2+^ or prevention of its uptake by mitochondria) resulted in the complete recovery of the membrane potential (Figure 3) [18], indicating that the flickering mode was stopped and the integrity of the membrane was restored.

PA/Ca^2+^-induced membrane permeabilization was also observed in the experiments with SRB-loaded erythrocytes. As can be seen in Figure 1d, the addition of PA did not result in a significant release of SRB from the cells. However, the subsequent addition of Ca^2+^ triggered a rapid release of the dye [20]. 

The observations of PA/Ca^2+^-induced membrane permeabilization on various membrane systems, model and biological, raise the question: are the molecular mechanisms of permeabilization the same in all these cases?

To address this question, we compared characteristics of PA/Ca^2+^-induced permeabilization of artificial and biological membranes—and it turned out that there were some similarities. Permeabilization of both liposomal and mitochondrial membranes was demonstrated to be inhibited by Ca^2+^-binding agents (EGTA and bovine serum albumin (BSA)) [18,21], as well as by cholesterol, a modulator of phase state properties of lipid bilayers [22]. Furthermore, liposomes enriched with the mitochondrial lipid cardiolipin were more easily permeabilized, and neutralizing cardiolipin in mitochondria with nonyl acridine orange inhibited permeability transition in the organelles [23,24]. These data suggest that the nature of membrane permeabilization is probably similar in the two types of membranes. They also indicate that the formation of the PA/Ca^2+^ complexes is a key event in the mechanism of PA-mPT.

## 4. Molecular Mechanism of PA-mPT

As stated above, we hypothesized that PA-mPT was caused by the formation of complexes between PA and Ca^2+^. The ability to bind Ca^2+^ with high affinity is a unique feature of long-chain saturated fatty acids (PA and SA (stearic acid)); other lipids and fats have much lower affinities to Ca^2+^ (Table 1) and cannot induce Ca^2+^-dependent permeabilization of model and biological membranes [16,19,20,21].

**Figure 4:**
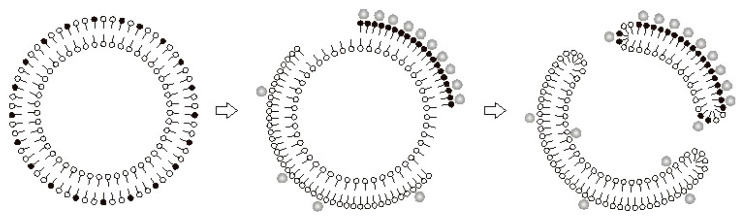
Schematic representation of hydrophilic lipid pore formation in the liposome membrane during a chemotropic phase transition. Adapted from Agafonov et al. J. Membrane Biol. 215 (2007): 57–68, doi:10.1007/s00232-007-9005-4.

We believe that permeabilization of membranes upon binding of Ca^2+^ to the anions of long-chain saturated fatty acids is related to a phase transition in the lipid bilayer (Figure 4). This interpretation was supported by the experiments with nonyl acridine orange (NAO), a self-quenching fluorescent probe which can be used to detect changes in the area of liquid phase of the lipid bilayer. Incorporation of PA molecules into liposomal membranes caused their expansion and a corresponding increase in the fluorescence intensity of NAO. The subsequent addition of Ca^2+^ decreased NAO fluorescence, suggesting a segregation of PA/Ca^2+^ complexes into solid-phase membrane domains [15]. 

The phase separation induced by Ca^2+^ in PA-containing membranes can be accompanied by the formation of pores in the lipid bilayer. The mechanism of pore formation under such “chemotropic” phase transition was described earlier by Antonov et al. [25,26]. In respect to PA and Ca^2+^, the mechanism can be described as follows. The binding of Ca^2+^ to PA anions at one side of a membrane will cause segregation of PA/Ca^2+^ complexes into solid-phase domains (Figure 4). Since solid-phase domains are more tightly packed, the overall area of the monolayer will be reduced. Thus, the monolayer containing solid-phase domains will become expanded, and the opposing monolayer will be condensed (stage 2; Figure 4, middle panel). At a certain point, the expanded monolayer may break, and the hydrophobic parts of the bilayer will be exposed to the aqueous phase, producing a highly unstable structure. Consequently, the condensed monolayer would also break and then fuse with the expanded monolayer, closing the hydrophobic edges of the rupture. The resulting structure will be a hydrophilic lipid pore (stage 3; Figure 4, right panel) [27,28,29].

Formation of hydrophilic lipid pores under the conditions when the bilayer is destabilized or stretched is a well-known phenomenon: the pores can be formed, for example, in the process of temperature-induced phase transition, as a result of osmotic shock, or can be produced by the electroporation technique [30,31]. One of the most important properties of lipid pores is their ability to spontaneously close after a short period of time (fractions of a second). In the case of PA and Ca^2+^, the diameter of such transient pores is approximately 4 nm, which is slightly bigger than the diameter of the classical mPTP, whose size is believed to be in the rage of 2–3 nm [16,19]. Moreover, unlike lipid pores, mPTP is believed to be a channel which can remain open for quite a long time. Furthermore, the formation of PA-mPT pores can be inhibited by RR, which does not affect the probability of mPTP opening [17,18]. The fact that mitochondria can keep operating for a while after PA-mPT is triggered may be, in our opinion, a key feature which enables regulation of many physiological and pathological processes, including apoptosis.

## 5. Properties of PA-mPTP and Its Regulation

When mitochondria are incubated in the presence of PA, the addition of Ca^2+^ causes permeability transition that is not inhibited by CSA (Figure 1c) [17,19]. These observations might appear in contrast to the previous reports that PA can potentiate the opening of the classical CSA-sensitive pore. Traditionally, the stimulatory effect of PA on mPTP was explained by either interaction of PA with the protein complexes of the inner mitochondrial membrane or its protonophore activity [32]. However, all those experiments were conducted in the presence of other mPTP activators (namely, orthophosphate and oxidants), which makes it difficult to estimate the specific contribution of PA to the process. 

PA-mPT demonstrates properties that distinguish it from the CSA-dependent mPT. First of all, the formation of PA-mPT pores requires much lower concentrations of Ca^2+^ compared to the classical mPT [13,19,33]. Albeit higher than the basal level of intracellular Ca^2+^, these concentrations are within the range of the pathological concentrations of intracellular Ca^2+^ observed during stress [34]. 

In our experiments, PA/Ca^2+^-dependent mitochondrial swelling was induced when PA was added at the amount of 30 nmol/mg protein [21]. The most common free fatty acids of the inner mitochondrial membrane are palmitic, stearic, and oleic (6.61, 6.46, and 5.06 molar % of the total lipids, respectively). The combined content of long-chain saturated free fatty acids (PA + SA) in the intact mitochondrial membrane is, therefore, lower than the content necessary to trigger permeability transition (it is estimated to be about 17–18 molar %). Under stress conditions, however, the content of free fatty acids in the mitochondrial membrane can be increased. 

The increase in the content of free fatty acids in cellular membranes under stress and pathological conditions is known to be related to the elevation of the Ca^2+^ concentration inside the cell. Ca^2+^ activates Ca^2+^-dependent phospholipases, including mitochondrial phospholipase A_2_ (PLA2) [35]. PLA2 mostly hydrolyzes phospholipids at the 2nd carbon atom of glycerol, which is primarily esterified by arachidonic acid—yet the enzyme is also capable of cleaving lipid molecules at the 1^st^ position, which is often occupied by saturated fatty acids [33,36]. Correspondingly, the activation of PLA2 by Ca^2+^ and/or oxidative stress leads to the release of various fatty acids, including PA [36,37,38].

There are some interesting facts about the cell lines that have increased contents of PA and SA (in both free and esterified forms). Specifically, WEHI-164 cells, which are highly prone to apoptotic cell death, are found to have an increased content of PA and SA as compared to the apoptosis-resistant C6 cells [17]. We have also found that, unlike common Wistar rats, the stress-resistant animals of the August line are much more susceptible to the formation of PA-mPT pores (particularly, at Ca^2+^ concentrations of 2.5–10 µM) [39]. 

## 6. Differences between PA-mPT and Classical mPT

PA-mPT and mPT seem to have different molecular mechanisms—and their properties, as well as regulation, also differ. 

As stated above, PA-mPT is not inhibited by CSA, a well-known inhibitor of mPT. Similarly, some other mPT modulators, including inhibitors Adenosine di-phosphate (ADP) and 2,4-Dinitrophenol (DNP) and activators Atractyloside (ATR), orthophosphate, and temerasol, do not affect the probability of PA-mPT [19]. 

Furthermore, unsaturated fatty acids, which are known to facilitate mPT, cannot induce the emergence of PA-mPT pores. Right the opposite, their presence in the mitochondrial membrane reduces the probability of PA-mPT [24,33].

Another condition necessary for the emergence of PA-mPT pores is the accumulation of Ca^2+^ in the mitochondrial matrix. Prevention of Ca^2+^ uptake by mitochondria—by the blockage of Mitochondrial Calcium Uniporter (MCU) with RR or by membrane depolarization—suppresses PA-mPT [18,19]. Thus, membrane potential is necessary for the emergence of PA-mPT pores, whereas mPTP opening can be triggered by membrane depolarization. Additionally, PA-mPT can be induced not only by Ca^2+^, but also by Sr^2+^ and, to a lesser extent, by Ba^2+^ [29]. All these ions can be transported into the mitochondrial matrix by MCU. This also sets PA-mPT apart from mPT, which can only be triggered by Ca^2+^. 

One of the most important properties of lipid pores is their ability to close spontaneously if their diameter does not exceed a certain critical value. For a liquid bilayer phase, this value was estimated to be ~9 nm. The closure of lipid pores occurs rapidly (they exist for a fraction of a second) and restores the integrity of the lipid bilayer [26]. PA-mPT pores are an example of such transient pores. The size of PA-mPT pores is estimated to be ~4 nm, which is slightly larger than the size of mPTP (~2–3 nm). The rapid closure of PA/Ca^2+^-induced lipid pores was demonstrated in the experiments with liposomes. On the other hand, rapid closure is not something usually observed in the case of mPTP, which often remains open for an extended period [16,19]. We suggest that the ability of PA-mPT pores to close spontaneously and rapidly underlies the so-called “flickering” mode of mitochondrial permeability transition, which is reversible and seems to play an important role in the regulation of many physiological and pathological processes, such as the emergency release of mitochondrial Ca^2+^ or induction of apoptosis [40].

## 7. The Mechanism of Formation of PA-mPT Pores

**Figure 5:**
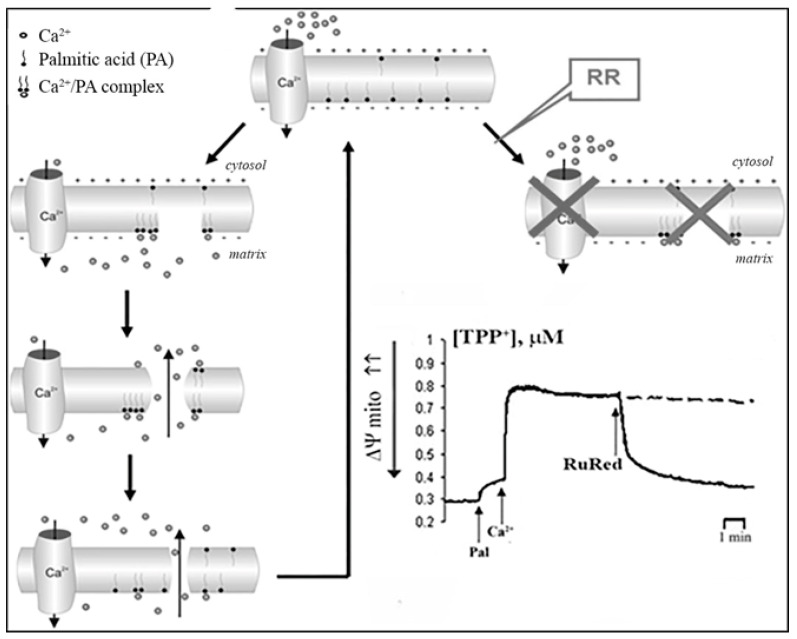
A possible mechanism of PA-mPT pore formation upon the activation of phospholipase A_2_.

Based on our experimental data, the putative mechanism of PA-mPT pore formation involves several steps (Figure 5). First, elevation of the intracellular concentration of Ca^2+^ leads to its enhanced uptake by mitochondria. Second, the accumulation of Ca^2+^ in the mitochondrial matrix results in the activation of PLA2 and raises the content of free fatty acids in the mitochondrial inner membrane. Third, the anions of long-chain saturated fatty acids form complexes with Ca^2+^ on the matrix side of the inner mitochondrial membrane and when their content reaches a certain threshold, they segregate into solid domains, which is accompanied by the formation of lipid pores.

The formation of pores leads to the mitochondrial depolarization and redistribution of ions across the mitochondrial membrane (in particular, the release of Ca^2+^ and reuptake of H^+^ by the organelles). Following the rapid closure of pores, membrane potential quickly recovers, owing to enhanced operation of the respiratory chain. Membrane repolarization leads to the reactivation of Ca^2+^ uptake and induction of a new uptake/release cycle.

**Figure 6:**
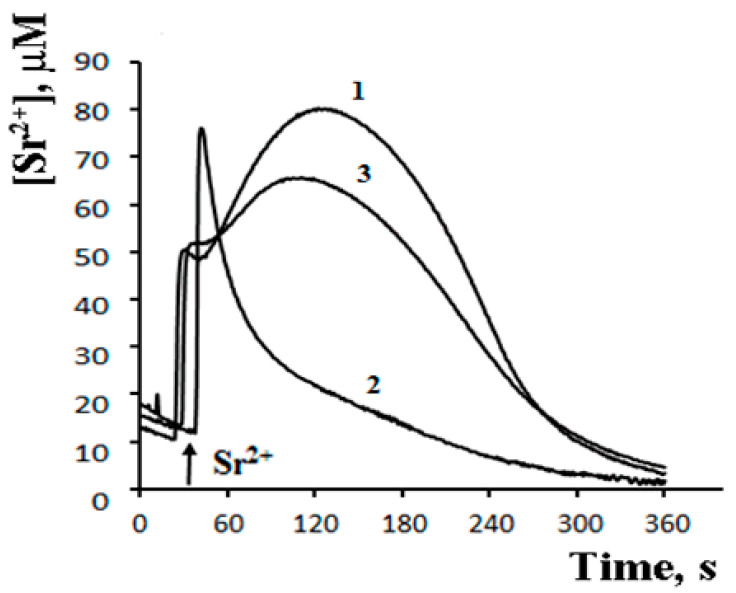
Palmitic acid re-induces the Sr^2+^-induced permeability transition suppressed by the Ca^2+^-dependent phospholipase A_2_ inhibitor—AACOCF_3_. The medium contained 20 mM sucrose, 1 mM KCl, 1 μM TPP^+^, 1 μM CsA, 5 mM succinic acid/Tris (pH 7.3). Addition: 40 nmol SrCl_2_/mg of protein. 1—control; 2—mitochondria preincubated with 15 μM AACOCF_3_; 3—mitochondria preincubated with 15 μM AACOCF_3_ and 40 μM PA. The concentration of the mitochondrial protein was 2 mg/mL. Typical curves are shown (*n* = 4). Adapted from Mironova et al. Biochim. Biophys. Acta. 1848 (2015): 488–495.

The possibility of such a cycle was demonstrated in the experiments with inhibitors of Ca^2+^-dependent PLA2 on a model of reversible Sr^2+^ uptake/release by mitochondria (Figure 6). A stepwise addition of Sr^2+^ to the suspension of energized rat liver mitochondria in a hypotonic medium (hypotonia was required for the activation of PLA2) was shown to cause a nonselective and reversible increase in the membrane permeability to Sr^2+^, H^+^, and K^+^ [41,42,43]. The addition of Sr^2+^ also led to a reversible membrane depolarization and CSA-insensitive mitochondrial swelling. We hypothesized that the observed changes were associated with the emergence of non-selective pores, following the formation of complexes of PA and SA with Sr^2+^ [44]. All these effects were prevented by aristolochic acid, an inhibitor of PLA2, further supporting the idea that the free fatty acids released by PLA2 are involved in the permeability transition. Notably, the addition of PA reversed the inhibitory effect of aristolochic acid and restored ion cycling across the membrane (Figure 6), emphasizing the key role of long-chain saturated fatty acids in the mechanism of membrane permeabilization. 

**Figure 7:**
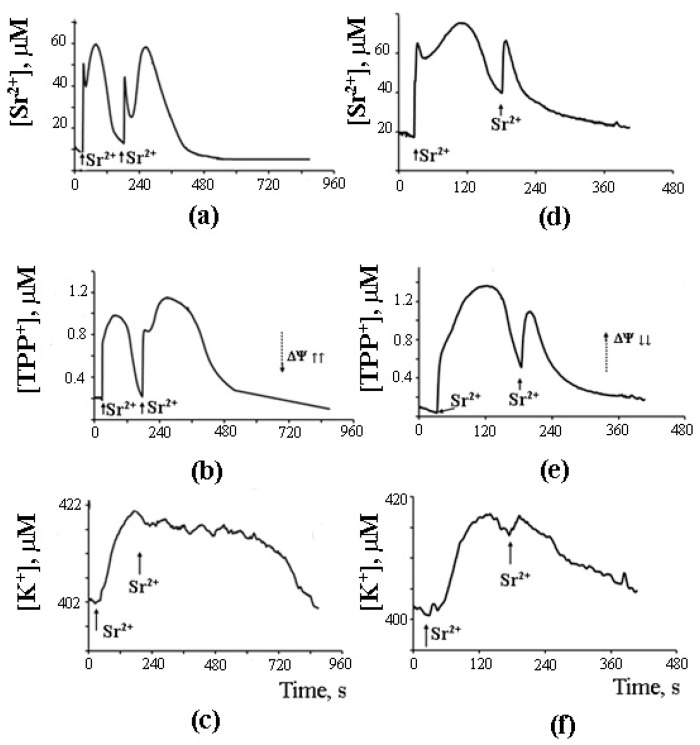
Sr^2+^-induced cyclic changes in the external concentrations of Sr^2+^, TPP^+^, and K^+^ ions in the suspension of rat liver mitochondria in the absence (**a**–**c**) or presence (**d**–**f**) of 15 μM AACOCF_3_ (phospholipase A_2_ inhibitor). Addition: 47 nmol SrCl_2_/mg of the protein. The medium was the same as in Figure 6. The concentration of the mitochondrial protein was 2 mg/mL. Typical curves are shown (*n* = 6). Adapted from Mironova et al. Biochim. Biophys. Acta. 1848 (2015): 488–495.

As can be seen in Figure 7, after the completion of a Sr^2+^ uptake/release cycle, mitochondria are functionally active, and the cycle can be repeated again in response to another Sr^2+^ pulse. 

What is important, though, is that the second cycle cannot be triggered before the first cycle has been completed. Apparently, there is some kind of “refractory” period, during which mitochondria are restoring their functional state [44]. A similar observation was made earlier by Holmuhamedov et al. [43]. 

## 8. A Possible Role of PA-mPT in the Emergency Release of Ca^2+^ from Mitochondria and Maintenance of Ion Homeostasis

On the basis of the experimental data obtained, we propose that PA-mPT pores play a protective role, releasing excessive Ca^2+^ from mitochondria—and thus can be called “pores of life.” As outlined in the previous section, a pulse of Sr^2+^ added to mitochondria in the presence of CSA triggers prolonged oscillations of functional parameters of the organelles: K^+^ and Sr^2+^ fluxes, membrane potential, pH, matrix volume, rate of oxygen consumption, and H_2_O_2_ production. These oscillations can be blocked by the inhibitors of Ca^2+^-dependent PLA2 (Figure 7). The fact that the oscillatory mode can be maintained for quite a long time suggests a possibility of similar oscillatory processes occurring in vivo, where they could be synchronized with the oscillations of cellular Ca^2+^. It is also possible that the formation of PA-mPT pores in such a “flickering” mode protects mitochondria from the pathological Ca^2+^ overload and prevents the permanent opening of CSA-sensitive mPTP.

Thus, PA-mPT forms a system of rapid Ca^2+^ release and H^+^ uptake, which is triggered in emergency situations accompanied by Ca^2+^ overload of mitochondria. It is also possible that the system may operate, to some extent, under normal circumstances, serving the same purpose as K^+^/H^+^ and Ca^2+^/H^+^ exchangers: maintaining ion homeostasis in the cell. 

The ideas that fatty acids can be involved in the release of Ca^2+^ from mitochondria and that this process is regulated by PLA2 were proposed over 40 years ago [45,46,47,48]. We continued developing these ideas in our works, considering the mechanism of membrane permeabilization in more detail and from a different perspective. We also obtained new evidence of the physiological role of the mechanism—in particular, observations that the susceptibility of mitochondria to the formation of PA-mPT pores is tissue-dependent and changes with age [49]. Another interesting observation was made towards hypoxia-resistant rats: their mitochondria turned out to be more susceptible to the permeabilizing effect of PA and Ca^2+^ as compared to the organelles of the control animals [50]. In other words, the resistance of animals to hypoxia seems to be associated with the ability of their mitochondria to form PA-mPT pores. This supposition was also supported by the results of measurements of H_2_O_2_ production and membrane potential. 

It should be noted that the classical mPTP was also proposed to have a flickering mode of operation and thus plays a protective role [51]. Indeed, a transient opening of mPTP should be sufficient to induce Ca^2+^ release from mitochondria followed by the restoration of their normal function. On the other hand, the lipid pore mechanism might be advantageous in this respect to the protein pore mechanism. First, lipid pores close spontaneously, which ensures that the membrane loses its integrity only for a short time. Further, the involvement of PLA2 guarantees that the lipid pore mechanism is strictly Ca^2+^-dependent [52,53]. Finally, a high rate of phosphorylation favors the formation of PA-mPT pores [39,50], which makes the lipid pore mechanism physiologically relevant. Overall, the involvement of mPT and PA-mPT in maintaining mitochondrial and cellular Ca^2+^ homeostasis requires further investigation.

## 9. The Mechanism of PA-mPT-Mediated Protection against Ca^2+^ Overload and mPTP Activation

**Figure 8:**
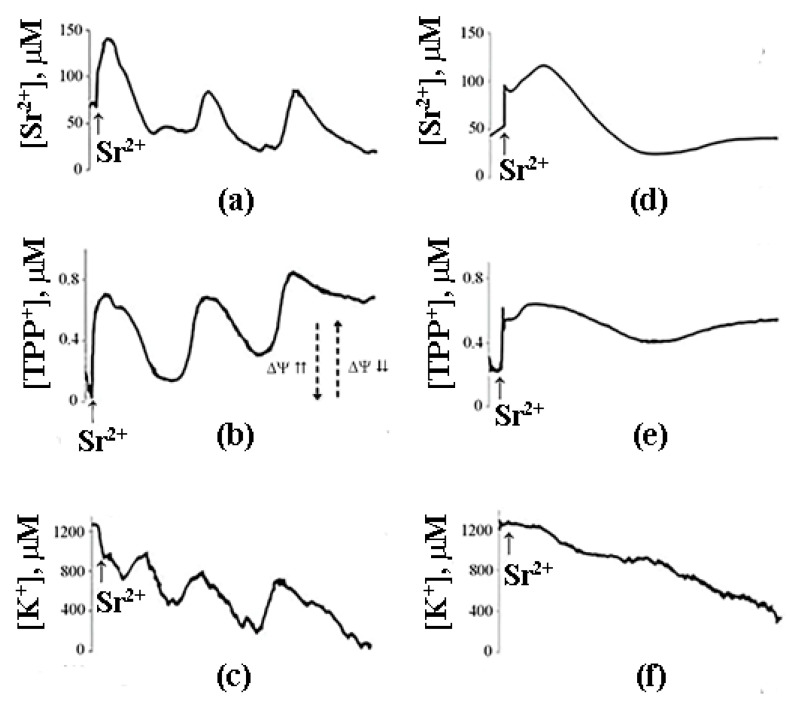
Sr^2+^/valinomycin-induced spontaneous changes in the external concentration of Sr^2+^, TPP^+^, and K^+^ ions in the suspension of rat liver mitochondria in the absence (**a**–**c**) or presence (**d**–**f**) of the inhibitor of Ca^2+^ (Sr^2+^)-dependent phospholipase A_2_, aristolochic acid (25 μM). Addition: 45 nmol SrCl_2_/mg mitochondrial protein. The medium was the same as in Figure 5 but in the presence of 1 ng/mg valinomycin/protein. The concentration of the mitochondrial protein in the cuvette was 2 mg/mL. Typical curves are shown (*n* = 7).

As mentioned above, the prolonged oscillations of ionic fluxes induced by Sr^2+^ in the presence of valinomycin were prevented by the inhibitors of Ca^2+^-dependent PLA2 (Figure 8). The involvement of PLA2 suggests that the release of ions from mitochondria occurs via PA-mPT pores. Their formation would equilibrate ionic gradients across the mitochondrial membrane. Notably, the inhibitors of Ca^2+^-independent PLA2 did not affect the oscillations. 

One of the features of PA-mPT is that the formation of lipid pores can be induced by a relatively small amount of Ca^2+^. Therefore, PA-mPT pores may start appearing in the mitochondrial membrane before the organelles become overloaded with Ca^2+^ to the extent when the opening of mPTP would be triggered. 

This supposition was supported by the comparative studies of mPT and PA-mPT in rats with genetically altered resistance to hypoxia. The experiments showed that in hypoxia-resistant animals, mitochondria were more susceptible to PA-mPT, as compared to the organelles of hypoxia-sensitive rats [50]. At the same time, the activation of mPTP in the resistant animals required much higher amounts of Ca^2+^. In addition, the resistant animals had a higher Oxidative Phosphorylation (OXPHOS) efficiency and lower rates of ROS production. All these observations were consistent with the idea that formation of PA-mPT pores protected mitochondria against Ca^2+^ stress and mPTP opening. 

The results obtained in the experiments with genetically altered animals were further confirmed in the studies of two lines of rats (August and Wistar) with different levels of the OXPHOS. It is established that out of these two lines, August rats are less sensitive to hypoxia and stress [54]. We found that August rats had higher respiration rates and OXPHOS efficiency, as well as higher rates of potassium fluxes and ATP-sensitive mitochondrial swelling and lower rates of H_2_O_2_ production. PA-mPT pores formed more easily in August rats, whereas mPTP required more Ca^2+^ to open [39]. Thus, these results confirmed the conclusion that PA-mPT provides some protection against Ca^2+^ overload of mitochondria. This supposition can also explain the effects of PLA2 inhibitors on mPTP opening, which were documented by Pfeiffer’s group in 2006 [55]. 

In summary, investigation of PA-mPT may help to understand the molecular mechanisms involved in the protection of mitochondria against Ca^2+^ overload, mPTP opening, and cell death. Importantly, the formation of PA-mPT pores does not uncouple OXPHOS in mitochondria, allowing the organelles to maintain energy supply during stress.

## 10. A Possible Role of PA-mPT in the Glutamate-Induced Neurotoxicity

One of the pathologies in which PA-mPT may play an important protective role is brain injury, when neurons become exposed to a prolonged and thereby toxic effect of glutamate. The mechanism of such glutamate-induced neurotoxicity is not completely understood [56]—although it is clear that the prolonged binding of glutamate to NMDA (N-Methyl-D-aspartic acid or N-Methyl-D-aspartate) receptors results in a severe Ca^2+^ stress (or so called delayed Ca^2+^ dysregulation, DCD) leading to the Ca^2+^ overload of mitochondria and, eventually, cell death. 

Initially, it was believed that the glutamate-induced damage to neurons is irreversible [57]. However, later studies demonstrated that the pathological process could be reversed (at least at early stages)—by inhibition of NMDA receptors or chelation of Ca^2+^ in the media [56]. Evidently, the mechanism of glutamate-induced neurotoxicity involves permeability transition in mitochondria, yet it is not easy to link it to the classical mPT. The replacement of Ca^2+^ (an essential activator of mPTP) with Sr^2+^ does not affect glutamate-induced neurotoxicity [58]. Furthermore, the effect of glutamate is not sensitive to CSA, a potent inhibitor of mPTP. Therefore, the mechanism of mitochondrial permeabilization under the conditions of glutamate neurotoxicity may be mPTP-independent. 

According to out supposition, the glutamate-induced Ca^2+^ dysregulation leads to the activation of PLA2, which is followed by the increase in the content of free fatty acids in the inner mitochondrial membrane and the formation of PA-mPT pores. This supposition was confirmed in the experiments with isolated rat brain mitochondria and cultured neurons. The experiments with neuronal mitochondria showed that Sr^2+^ induced a CSA-insensitive permeabilization of the mitochondrial membrane, which was accompanied by the release of the cation, a drop in the membrane potential, and swelling of the organelles. All these effects were blocked by the inhibitors of Ca^2+^-dependent PLA2, suggesting the involvement of PA-mPT pores in the mechanism of membrane permeabilization [44]. At the same time, the effects were insensitive to the inhibitors of mPTP. On the other hand, BSA, which binds free fatty acids, prevented membrane permeabilization. 

**Figure 9:**
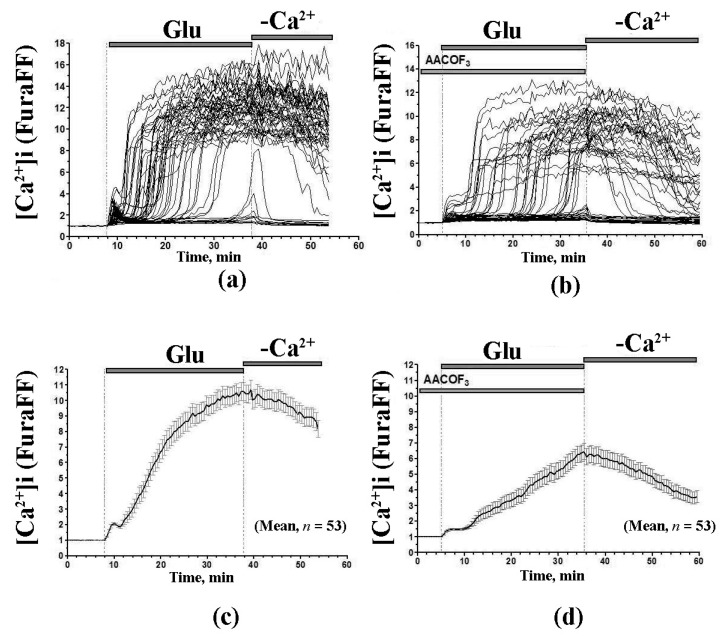
Inhibition of PLA2 increases the latent period of DCD in the granular neurons of rat cerebellum. The effect of Glu (100 μM) on the dynamics of [Ca^2+^]_i_ in the neuron culture in the absence (**a**,**c**) or presence (**b**,**d**) of the PLA2 inhibitor AACOCF3. (**a**,**b**) Traces of [Ca^2+^]_i_ changes in the individual neurons; (**c**,**d**) averaged changes of [Ca^2+^]_i_ for the same neurons. Changes of [Ca^2+^]_i_ are expressed as the ratio of FuraFF fluorescence excited at 340 and 380 nm (F340/F380). The mean values of [Ca^2+^]_i_ were averaged for 53 neurons. AACOCF3 (10 μM) was added one hour before the Glu exposure. Adapted from Mironova et al. Biochem. Mosc. Suppl. S, 6 (2012): 45–55.

The experiments with cultured neurons demonstrated that inhibitors of Ca^2+^-dependent PLA2 also suppressed the glutamate-induced changes in the intracellular concentration of Ca^2+^ [59]. More specifically, the addition of glutamate to cultured neurons first leads to a spike of intracellular Ca^2+^, which is followed by the secondary (delayed) elevation of the intracellular cation concentration. The addition of PLA2 inhibitors significantly delayed the secondary elevation of intracellular Ca^2+^ (Figure 9). These inhibitors also prevented mitochondrial depolarization [59]. 

On the basis of these data, we proposed that the glutamate-induced elevation of intracellular Ca^2+^ stimulates the formation of PA-mPT pores, which helps to prevent mitochondrial Ca^2+^ overload and, possibly, mPTP opening. The putative mechanism is outlined in the previous sections: activation of PLA2 increases the content of free fatty acids in the mitochondrial membrane, and formation of PA/Ca^2+^ complexes on the matrix side of the membrane followed by their segregation would be accompanied by the emergence of transient lipid pores [17,19]. 

It should be noted that among the major fatty acids released by PLA2 would be not only PA and SA, but also arachidonic acid [33]. Although arachidonic acid does not participate in the phase-transitional mechanism of membrane permeabilization, it might play an important role at the late stages of Ca^2+^ dysregulation, which include apoptotic cell death [56,60]. 

The formation of PA-mPT pores might also underlie the glutamate-induced apoptosis and necrosis. Indeed, there are reports that PA and SA are able to stimulate apoptosis [61]. We found that prolonged permeabilization of the mitochondrial membrane caused by high concentration of PA leads to the release of CytC and apoptosis Inducing Factor (AIF) from mitochondria [23,49]. It is possible that formation of lipid pores in the membrane of neuronal mitochondria at irreversible stages of glutamate-induced Ca^2+^ dysregulation might trigger the release of proapoptotic proteins from the organelles. If the program of cell death is launched when PA-mPT operates in the “flickering” mode, it might be beneficial to the cell, since mitochondria will still be functional, supplying energy for the execution of the apoptotic cascade.

## 11. Conclusions

In conclusion, the data of literature and our works indicate that, in certain cases, permeability transition in mitochondria can be triggered by the formation of transient, CSA-insensitive pores (PA-mPT pores), which are fundamentally different from the classical Ca^2+^-induced CSA-sensitive mPTP. Formation of PA-mPT pores can be induced by moderate amounts of Ca^2+^, and they can emerge in mitochondria with high phosphorylating activity. Hence, PA-mPT may play an important protective role in the cell, preventing Ca^2+^ overload of mitochondria and, perhaps, participating in the adaption of the organelles to stress. 

Speaking about perspectives of PA-mPT research, it would be particularly important to continue studying different modes of mitochondrial permeabilization. If we can separate the transient, flickering mode of permeabilization (the “pore of life”) from the permanent permeabilization mode (the “pore of death”)—by identifying conditions for their triggering and expanding the arsenal of pharmacological agents to control them—it may be very useful in the therapy of many pathologies. 

Further studies are needed to get more detailed information about the role of PA-mPT in the mitochondrial physiology and protection of the organelles and the cell against various kinds of stress.

## Figures and Tables

**Table 1:** Binding of various fatty acids and lipids ^1^.

Lipids	Relative Ca^2+^ Binding
Lauric acid (12:0)	0.50 ± 0.03
Myristic acid (14:0)	7.30 ± 0.25
Palmitic acid (16:0)	83.00 ± 0.75
Stearic acid (18:0)	100.00
Eicosanoic acid (20:0)	73.00 ± 2.5
Docosanoic acid (22:0)	44.00 ± 1.2
Lignoceric acid (24:0)	15.00 ± 0.35
Palmitoleic acid (16:1)	1.90 ± 0.08
Oleic acid	5.70 ± 0.12
Linoleic acid (18:2)	0.65 ± 0.04
Linoleinic acid (18:3)	0.87 ± 0.05
Arachidonic acid (20:4)	1.10 ± 0.05
1-Palmitoyl-lysophosphatidylcholine	0.43 ± 0.02
1-Stearoyl-lysophosphatidylcholine	0.47 ± 0.02
1-Lauroyl-lysophosphatidylcholine	0.43 ± 0.01
Lysophosphatidylserine	0.54 ± 0.03
1,2-Dipalmitoyl-sn-glycero-3-phosphatidylcholine	0.40 ± 0.2
1,2-Dipalmitoyl-sn-glycero-3-phosphatidylethanolamine	0.22 ± 0.01
1-Palmitoyl-sn-glycero-1-3-phosphatidylethanolamine	0.76 ± 0.03
Palmitoil-CoA	0.43 ± 0.02
Cardiolipin	0.60 ± 0.03
L-α-phosphatidic acid	19.50 ± 0.8
Cholesterol	0.33 ± 0.01
Cerebrosides	0.20 ± 0.01
Sphingomyelin	0.30 ± 0.01

^1^ Five microliters of 2 mM solution of each lipid were applied to a Polyvinylidene difluoride (PVDF) membrane and binding of Ca^2+^ to the sample was estimated at pH 8.5 in the presence of 5 µM^45^ CaCl_2_. Ca^2+^ binding was measured in counts per minute. Stearic acid was taken as a reference to calculate the relative Ca^2+^ binding. Data are expressed in percentage and as the means ± SD of four experiments.

## Data Availability

Not applicable.
